# Concurrent tuberculous chorioretinitis with choroidal neovascularization and tuberculous meningitis: a case report

**DOI:** 10.1186/s12886-020-01504-y

**Published:** 2020-06-12

**Authors:** Yan-Kun Zhang, Hong-Yi Fu, Yan Guan, Yu-Jing Li, Hong-Zhong Bai

**Affiliations:** 1 Department of Ophthalmology, Hebei Chest Hospital, Shijiazhuang, 050000 China; 2 Department of Tuberculosis, Hebei Chest Hospital, Shijiazhuang, 050000 China; 3 Clinical Laboratory, Hebei Chest Hospital, Shijiazhuang, 050000 China; 4 Department of Radiology, Hebei Chest Hospital, Shijiazhuang, 050000 China

**Keywords:** Intraocular tuberculosis, Tuberculous chorioretinitis, Tuberculous meningitis, Optical coherence tomography, Choroidal neovascularization, Vascular endothelial growth factor

## Abstract

**Background:**

Tuberculosis (TB) remains a severe health burden worldwide. The manifestation of concurrent tuberculous cerebral and ocular involvements associated with TB is uncommon.

**Case presentation:**

We report a 17-year-old girl with concurrent tuberculous cerebral and ocular involvements and visual impairment due to choroidal neovascularization. This study emphasizes the definite diagnosis with the combination of ophthalmological examination, multimodal imaging and routine tuberculosis testing, and the proper management with intravitreal anti-VEGF injection accompanied by systemic anti-tuberculosis therapy.

**Conclusion:**

Combined applications of routine TB tests, fundus multimodal imaging and diagnostic therapy greatly help the clinician to establish a precise diagnosis and in monitoring the therapeutic response.

## Background

Among 30 high tuberculosis (TB) burden countries, China has the second largest number of TB cases, accounting for approximately 9% of the world’s cases [1]. TB most commonly occurs in the lungs, but can also affect virtually all other organs, which is known as extrapulmonary TB. Intraocular TB is a unique form of extrapulmonary TB, with various incidences geographically [2]. Due to the absence of ocular biopsies and protean clinical manifestations, intraocular TB presents a diagnostic challenge. Fortunately, several advancing imaging techniques have realized in vivo near-histologic visualization of the pathologic and structural alterations, which can help the oculist to make an accurate diagnosis and monitor the therapeutic response of tubercular lesions [3]. Here, we describe a patient suffering from tuberculous meningitis and tuberculous chorioretinitis with choroidal neovascularization (CNV), and highlight the fundus imaging manifestations, anti-TB treatment and intravitreal anti-vasculature endothelial growth factor (VEGF) management.

## Case presentation

A 17-year-old female patient complained of blurred vision in her right eye (OD) for 4 months. Further questions about the medical history revealed that the patient presented with intermittent low fever, cough for 4 months and headache for 4 days. Physical examination revealed neck stiffness and bilaterally diminished breath sounds, with a temperature of 37.8 °C. On examination, visual acuity in the right eye (OD) was hand motion at 10 cm, and visual acuity in the left eye (OS) was normal. The intraocular pressure was 18 mmHg in the right eye and 16 mmHg in the left eye.

**Fig. 1:**
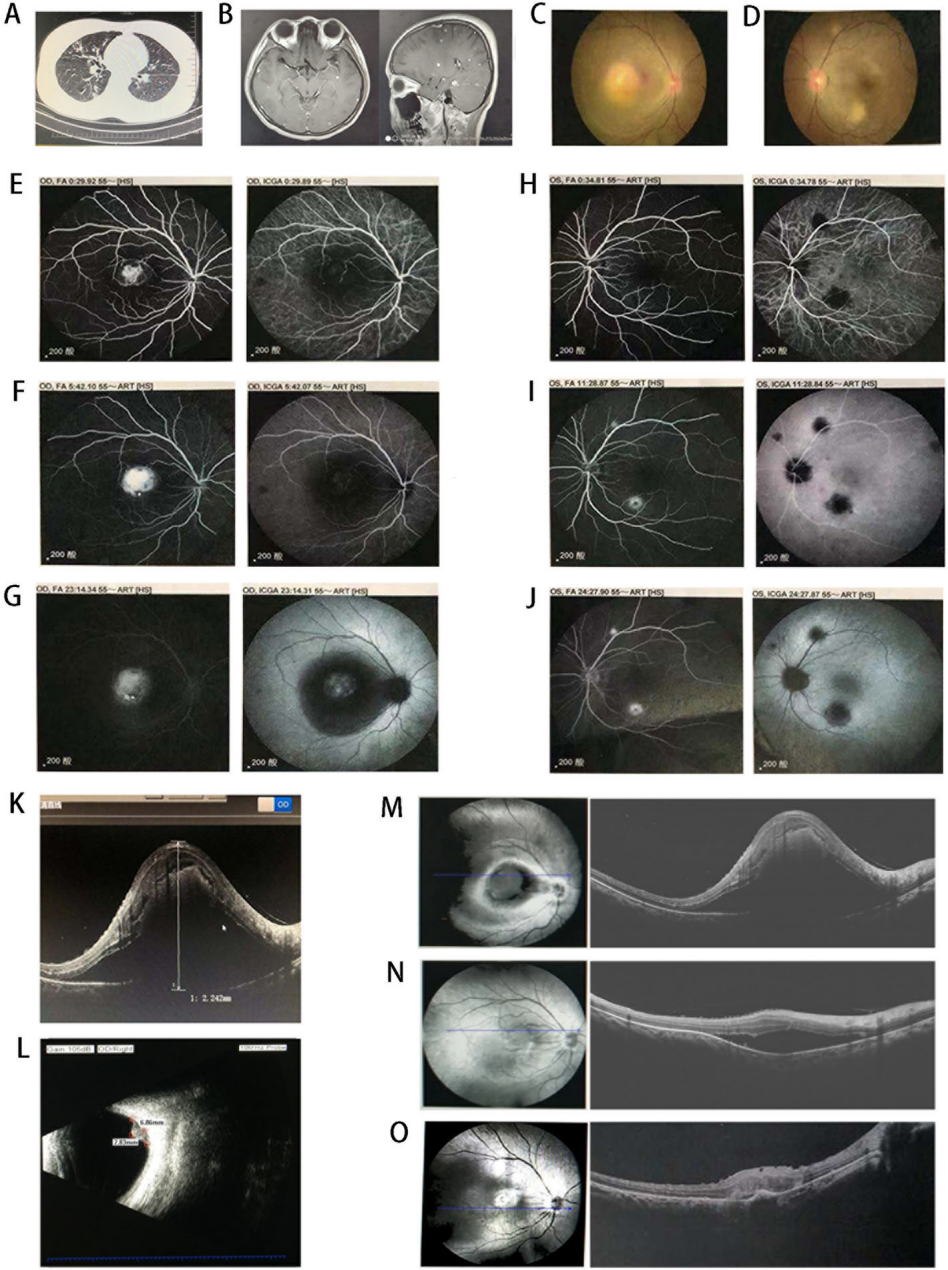
Chest CT demonstrating miliary nodules in the lung (**a**). Cerebral MRI displaying multiple intracranial nodules, and miliary nodule lesions with peripheral edema (**b**). Fundus photograph showing macular oedema, stellate exudation and an absent foveal reflex in the right eye (**c**), and sporadic yellowish-white exudation at the posterior pole in the left eye (**d**). Fundus fluorescein angiography and indocyanine green angiography in early (**e** and **h**), mid (**f** and **i**) and late (**g** and **j**) phases showing hyperfluorescence with leakage of dye from the core lesion in the right eye, and multiple lesions with early hypoautofluorescence and late hyperautofluorescence in the left eye. Optical coherence tomography displaying exudative retinal detachment (**k**). Ocular ultrasonography B-scan showing an oval solid mass in the posterior wall of the right eye (**l**). Optical coherence tomography showing exudative retinal detachment before intravitreal anti-VEGF treatment (**m**), and resolution of subretinal fluid at 1 week (**n**) and 1 month (**o**) follow-up after intravitreal ranibizumab injection

Chest CT showed miliary nodules in the lung (Fig. 1a). Cerebral MRI displayed multiple intracranial nodules, and diffused miliary nodule lesions with peripheral edema (Fig. 1b). The purified protein derivative (PPD) skin test, MycoDot test [4] and interferon-gamma release assay yielded positive results. Cerebral examination showed increased intracranial pressure, elevated cerebrospinal fluid (CSF) protein concentration, and decreased CSF glucose and chloride, suggesting the diagnosis of tuberculous meningitis [5]. Ocular fundus photography showed macular oedema, stellate exudation and an absent foveal reflex in the right eye (Fig. 1c), and sporadic yellowish-white exudation at the posterior pole in the left eye (Fig. 1d).

Simultaneous fundus fluorescein angiography (FFA) and indocyanine green angiography (ICGA) demonstrated early irregular hyper-fluorescence in macular center of the right eye, and sustained hyper-fluorescence with a ring of leaking surrounding the lesion on FFA and ICGA in the late phase (Fig. 1e-g), highly suspicious for choroidal granuloma with CNV. FFA showed two lesions as early hypoautofluorescent and late hyperautofluorescent with central hypoautofluorescence, and ICGA showed three hypofluorescent spots during the initial and transit periods with the late phase presenting hypofluorescent centers and surrounding hyperfluorescent edges in the left eye (Fig. 1h-j), suggesting the diagnosis of choroidal tubercles.

In the right eye, optical coherence tomography (OCT) revealed chorioretinal elevation of macular area, with an area of exudative retinal neurepithelium layer detachment (subretinal fluid sonolucent height was 2.242 mm) (Fig. 1k). Ocular ultrasonography B-scan revealed an oval solid mass measuring 6.86 mm × 2.83 mm in the posterior wall of the right eye (Fig. 1l).

The patient was started on anti-TB medications. She was treated previously with oral corticosteroids daily 30 mg for 1 month, and her symptoms of fever and fatigue disappeared, but her visual acuity of the right eye did not improve. In consideration of her well-defined TB with miliary spread, the patient received a six-month course of HRZE anti-tubercular regimen comprising isoniazid (0.3 g), rifampicin (0.45 g), pyrazinamide (1.5 g) and ethambutol (0.75 g) and intravenous dexamethasone (10 mg daily for 2 weeks and 5 mg daily for 2 weeks), followed by oral prednisone 30 mg daily with a slow taper. Meanwhile, the patient received an intrathecal isoniazid (0.1 g) and dexamethasone sodium phosphate (5 mg) injection OD.

Her systemic condition gradually improved during the first 10 days of combination therapy. Cerebral pressure gradually returned to normal from 240 mmH_2_O, CSF protein concentration decreased, and CSF glucose and chloride returned to normal levels (Table 1), supporting the diagnosis of tuberculous meningitis. However, the combination therapy did not improve macular edema in the right eye. Then, the patient received an anti-VEGF ranibizumab (0.05 mL) injection in the right eye. One month later, OCT scan showed clear retinal structure with a significant improvement of macular edema and disappearance of subretinal fluid (Fig. 1m-o). The final visual acuity was 0.2 in the right eye and 1.0 in the left eye.

**Table 1:** The effect of combination therapy of cerebral pressure and cerebrospinal fluid examination

	Pre-treatment	8 days	10 days
Cerebral pressure (mmH_2_O)	240	150	130
CSF protein (g/L)	1.18↑	0.58↑	0.55↑
CSF glucose (mmol/L)	2.11↓	3.25	3.02
CSF chloride (mmol/L)	115↓	122	123
CSF adenosine deaminase (U/L)	2.0	1.0	0.8

Annotation: ↑ represents elevated level, ↓ represents decreased level, CSF cerebrospinal fluid.

## Discussion and conclusions

Intraocular TB has a variety of clinical manifestations and may mimic other intraocular inflammatory diseases, making the diagnosis and management of some asymptomatic intraocular TB challenging [6]. Meanwhile, the acute lesions of chorioretinitis can be difficult to distinguish from CNV based on clinical examination and FFA. In current clinical practice, OCT has become an indispensable ancillary test in the diagnosis and management of retinochoroid lesions, which has been termed “optical biopsy”. OCT shows characteristic features of CNV, which can be very useful in distinguishing inflammatory and neovascular lesions in uveitis [7, 8]. Thus, it is strongly suggested to perform OCT scan for patients with disseminated TB to screen for ocular involvement.

The choroid is the most commonly affected ocular structure in intraocular TB [6, 9]. The diagnosis of tuberculous chorioretinopathy remains a challenge and is usually presumptive. Thus, several lines of evidence should support the diagnosis of tuberculous chorioretinitis, such as history of systemic or previous TB, *Mycobacterium tuberculosis* detected in body fluids or tissues, the characteristic fundus lesions, PPD positive, good response to anti-tuberculosis therapy, and exclusion of other systemic diseases causing choroidal inflammation.

Clinician presumptive diagnosis is considered as a promising first step toward the correct diagnosis of intraocular TB [10]. Meanwhile, the favorable clinical response to anti-tuberculosis therapy verifies the presumptive diagnosis in time. In this case, the systemic condition gradually improved during the first 10 days of HRZE anti-tubercular regimen, confirming the presumed diagnosis. Whereas, there was no significant improvement in visual acuity under the anti-tuberculosis therapy. Thus, anti-VEGF agent was administrated, which is now the main recommendation in the management of CNV. The lack of response to anti-tubercular treatment and anatomical response to anti-VEGF support this diagnosis of an inflammatory choroidal CNV. CNV tends to occur in the macular area in patients with intraocular TB, which is one of the manifestations of intraocular TB. Previous studies have described the manifestation of inflammatory choroidal CNVs in tubercular and other forms of uveitis, and proposed several therapeutic strategies for intraocular TB-related CNV [11, 12], of which anti-VEGF intravitreal injection is the mainstream in its management. Thus, for disseminated TB with intraocular TB-related CNV, it is recommended to implement intravitreal anti-VEGF injections accompanied by systemic anti-tuberculosis medications and oral corticosteroids [12].

Macular CNV has been identified to be associated with intraocular TB. It is important to recognize CNV as a potential sequela of TB-related chorioretinitis and reveal the underlying mechanism of CNV development. According to Agarwal et al. [11], TB-associated CNV is typically adjacent to the healed choroidal granuloma or healed choroiditis scar lesions. The suggested underlying mechanism is that retinal pigment epithelium can act as reservoir for *Mycobacterium tuberculosis* infection and can be the likely site for delayed reactivation and development of posterior uveitis [13]. Moreover, Kim et al. [12] retrospectively reported four cases with active CNV secondary to intraocular TB, in which CNVs were developed 3 ~ 6 months after anti-TB medication. In this case, intraocular TB represented the first manifestation of an underlying systemic infection, with co-occurrence of CNV development and pulmonary symptoms. Generally, intraocular TB has diverse clinical manifestations, either by an active infection or an immunological reaction. The co-occurrence of CNV development and pulmonary symptoms might be related to an immune-mediated hypersensitivity response. This case was a 17-year-old girl with normally functioning immune system, might resulting in a latent or dormant infection in early stage. She had a history of ocular involvements at least 4 months in the interrogation. Given the situation of delayed seeking medical care in China, her history of ocular involvements might be much longer, which provide time for the development of CNV.

This study highlights a case presenting with active CNV secondary to tuberculous chorioretinopathy and tuberculous meningitis. This patient was treated with systemic anti-tuberculosis therapy, oral corticosteroids, and intravitreal anti-VEGF injection, and received a favorable clinical response. Combined applications of routine TB tests, fundus multimodal imaging and diagnostic therapy greatly contribute the clinician to establishing precise diagnosis and monitoring the therapeutic response. Systemic anti-tuberculosis medications and corticosteroid therapy frequently result in unsatisfactory optical response, and concomitant intravitreal anti-VEGF injection is an optimal strategy for tuberculous chorioretinitis with CNV.

## Data Availability

All data generated or analyzed during this study are included in this published article.

## Abbreviations

TB: Tuberculosis
CNV: Choroidal neovascularization
VEGF: Vasculature endothelial growth factor
PPD: Purified protein derivative
CSF: Cerebrospinal fluid
FFA: Fundus fluorescein angiography
ICGA: Indocyanine green angiography
OCT: Optical coherence tomography
